# Anesthesia Providers’ Perspectives on the Redesigned Philips Acoustic Alarm System: Qualitative Pre- and Postimplementation Study

**DOI:** 10.2196/82703

**Published:** 2026-04-01

**Authors:** Greta Gasciauskaite, Stefanie Senn, Cynthia Hunn, Christoph B Nöthiger, David W Tscholl, Tadzio Raoul Roche

**Affiliations:** 1 Institute of Anaesthesiology and Perioperative Medicine, University Hospital Zurich, University of Zurich, Raemistrasse 100, Zurich, 8091, Switzerland, 41 432530255; 2 Clinic for Anaesthesiology, Winterthur Cantonal Hospital, Winterthur, Switzerland

**Keywords:** anesthesia, auditory perception, clinical alarms, equipment design, patient monitoring, patient safety, sound, user-centered design, human-computer interaction

## Abstract

**Background:**

Alarm fatigue caused by frequent or false alarms poses a persistent threat to patient safety. Despite technological progress, alarm acoustics remain largely unchanged and are often perceived as disruptive. To address this, Philips redesigned its patient monitoring alarm sounds through a user-centered approach aimed at improving priority differentiation and reducing emotional strain.

**Objective:**

This study provides insights into human-technology interaction by examining anesthesia providers’ experiences with the original and updated alarms, with a focus on emotional responses, usability, and guidance for the user-centered design of future clinical alarm systems.

**Methods:**

This single-center qualitative study involved anesthesia providers who completed an online questionnaire before and after the implementation of the updated Philips alarms. Only those who completed the pre-implementation phase participated in the postimplementation phase. The questionnaire included 4 open-ended questions addressing perceptions of the current alarm sounds, suggestions for improvement, design expectations, and attitudes toward an alarm-free operating room. Responses were analyzed using thematic analysis to identify key usability and emotional response themes.

**Results:**

A total of 90 eligible anesthesia providers participated in the preimplementation phase, and 77 (85.6%) participated in the postimplementation phase. Positive emotional responses increased in the postimplementation phase, whereas concerns regarding alarm functionality also became more prominent. Before the introduction of the updated alarm sounds, participants predominantly called for softer sounds. Following implementation, the most frequently expressed concern shifted to the need for clearer prioritization of alarms. Across both phases, the primary expectation remained the alarms’ ability to effectively capture attention. The concept of an alarm-free operating room elicited concerns about increased workload and potential risks to patient safety.

**Conclusions:**

The redesigned alarm sounds were perceived more positively in terms of emotional acceptance; however, they did not improve the recognition of alarm priority. The modest acoustic changes did not address the broader issue of alarm overload. Suggestions such as visual-only alerts for low-priority alarms show potential but must be balanced with patient safety standards. Future alarm development should combine user feedback with expert-driven and evidence-based approaches to improve both usability and clinical effectiveness.

## Introduction

Alarm sounds are essential safety mechanisms in modern clinical environments, designed to alert caregivers to potentially critical changes in a patient’s condition [1]. However, high exposure to alarms, particularly those that are clinically irrelevant or false positives, can lead to alarm desensitization—a phenomenon known as alarm fatigue [2]. This is associated with delayed or missed responses to true alarms and represents a significant threat to patient safety [3,4]. Beyond the sheer volume of alarms, the lack of clarity and contextual relevance of alarm sounds further exacerbates the problem [5]. Poorly differentiated alarm sounds or those lacking intuitive meaning may cause confusion among clinicians and impair the timely interpretation of patient status [6-8], particularly in already crowded acoustic environments where multiple devices compete for clinicians’ attention.

Despite significant advances in patient monitoring technology, the acoustic design of alarms has seen little innovation over the past decades, despite extensive research in this area [9-11]. Historically, alarm sounds were designed to be as loud and attention-grabbing as possible, often by using auditory roughness and very salient, high-pitched frequencies [12]. The goal was to ensure that clinical staff would notice the alarms immediately. However, clinical staff often perceive these sounds as disruptive and emotionally taxing, with studies reporting that they are frequently experienced as stressful, difficult to interpret, and hard to distinguish from one another [8,13-15]. However, in many cases, subtle cue tones may be more appropriate as not all alarms require immediate action by health care professionals.

Philips (Koninklijke Philips N.V.), a leading manufacturer of patient monitors, has recognized these issues. In response, the company initiated a user-centered redesign process to revise the auditory characteristics of its monitor alarms. The updated system introduced changes to the acoustic cues with the aim of improving the distinction between alarm priorities and optimizing the balance between attention-grabbing and pleasant auditory stimulation [16]. The underlying logic of when and why alarms are triggered remained unchanged.

The redesigned alarm sounds attempt to address issues of poor clarity, limited contextual relevance, and negative emotional responses. However, it remains unclear whether such user-centered design changes translate into meaningful improvements in clinical practice. While the inclusion of end users in the design process is crucial, it may also limit innovation if feedback is primarily shaped by existing habits and expectations rather than by exposure to novel alarm paradigms [17]. More progressive approaches to alarm sonification, such as auditory icons or spoken cues that are easily distinguishable and convey information about the underlying cause of the alarm in addition to attracting attention, do not appear to have been considered in this redesign [9,10,18].

This qualitative study explored anesthesia providers’ perceptions of patient monitoring alarms before and after the implementation of the redesigned Philips sounds. Specifically, we investigated emotional responses, perceived usability, and participants’ visions for future alarm systems while reflecting on the broader implications for alarm design, innovation strategies, and patient safety.

## Methods

### Ethical Considerations

The Cantonal Ethics Committee Zurich reviewed the study protocol and issued a declaration of nonjurisdiction (Req-2022-00689) [19]. Before participation, written informed consent was obtained from all participants, granting permission for the use of the collected data for research purposes. Participation in the study was voluntary and without compensation. All data were deidentified prior to analysis. The data are presented in aggregated form only, and no conclusions about individual participants can be drawn from the published results.

### Updated Philips Patient Monitor Alarm Sounds: Design and Implementation

While the design process behind the updated Philips alarm sounds was not part of this study, we briefly summarize its key objectives to provide context. The redesign followed a human-centered approach, combining qualitative methods (interviews and workshops) with quantitative surveys across 2 iterative development phases. During the original design process, anesthesia professionals (including nurses, residents, and attending physicians) from 7 countries contributed their perspectives on what constitutes an improved alarm sound.

Participants consistently highlighted the need to reduce the harshness and unpleasantness of alarm cues, expressing a preference for sounds that are more pleasant, natural, and less stressful. They also emphasized the importance of clearer differentiation between alarm priority levels and better distinction from other clinical acoustic signals.

In the second iteration, participants were asked to evaluate and select from a range of newly developed alarm sound options. On the basis of this feedback, the sounds were revised as follows: low-priority alarms were softened and spaced with longer tone intervals, medium-priority alarms were given distinctive pitch and timing characteristics, and high-priority alarms were adjusted to reduce shrillness while maintaining urgency. A more detailed description of the design process can be found in the publication by Hunn et al [20]. Both the original and updated alarm sounds are available for comparison in Multimedia Appendix 1.

### Study Design

This investigator-initiated, single-center qualitative descriptive study was conducted at University Hospital Zurich, Switzerland, in 2 phases: before (January 2024 to March 2024) and after (August 2024 to October 2024) the implementation of the updated Philips patient monitoring alarm sounds. The aim was to explore anesthesia providers’ (trainee nurses, nurses, resident physicians, and attending physicians) perceptions of the original and updated alarm sounds following their implementation and a subsequent adaptation period of 4 months.

Before implementation, clinical staff participated in a 1-hour training session to familiarize themselves with the new alarm system.

An online questionnaire consisting of both open- and closed-ended questions was completed by participants as part of a face-to-face study procedure. In the prephase, participants were eligible if they worked regularly in the operating room and were familiar with the existing alarm sounds; no additional inclusion or exclusion criteria were applied. Only participants who had taken part in the prephase took part in the postphase.

### Survey Questionnaire

The questionnaire was developed using Google Forms and administered to participants at 2 time points: once during the prephase (original Philips alarms) and again during the postphase (updated Philips alarms). It comprised 2 main sections.

The first section included 4 open-ended questions designed to elicit free-text responses regarding participants’ perceptions of current patient monitoring alarm sounds, suggested improvements and their underlying reasoning, expectations for alarm cue design, and vision of an operating room without acoustic alarms.

The second section contained 4 closed-ended questions to collect demographic data (age, gender, years of experience in anesthesia, and professional role) during both phases. In the prephase only, 2 additional questions were included to assess whether participants regularly played a musical instrument and whether they had attended an introductory lecture on the redesigned alarm sounds before their clinical implementation.

### Data Analysis

**Figure 1.**
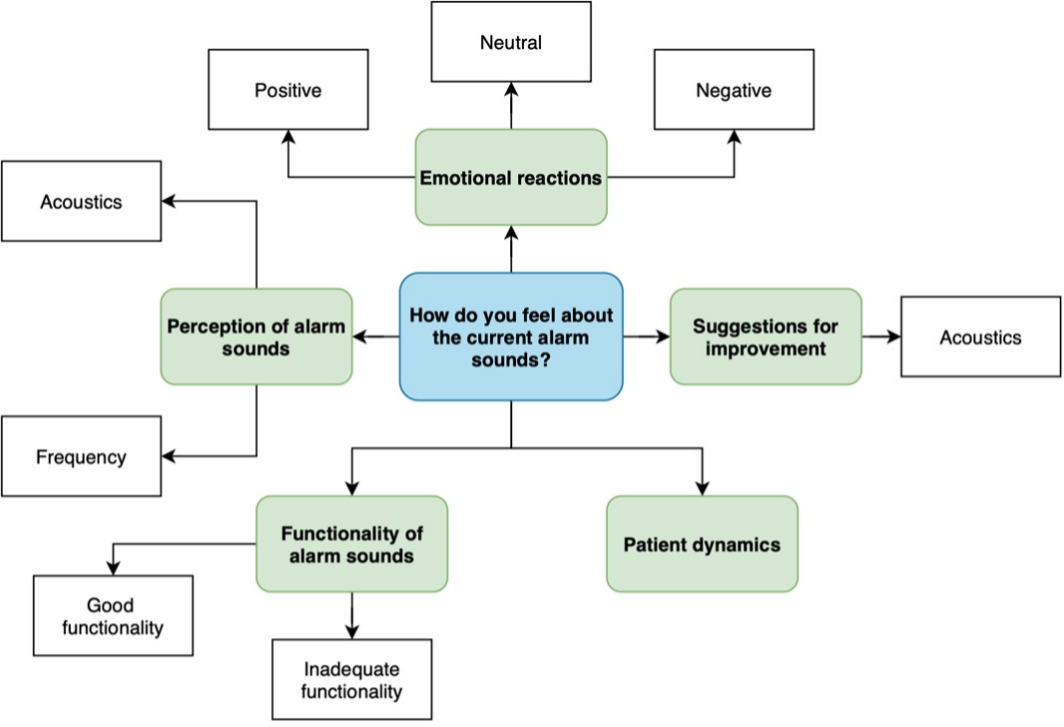
Coding tree illustrating the main themes and subthemes identified when participants were asked how they felt about the current alarm sounds.

**Figure 2.**
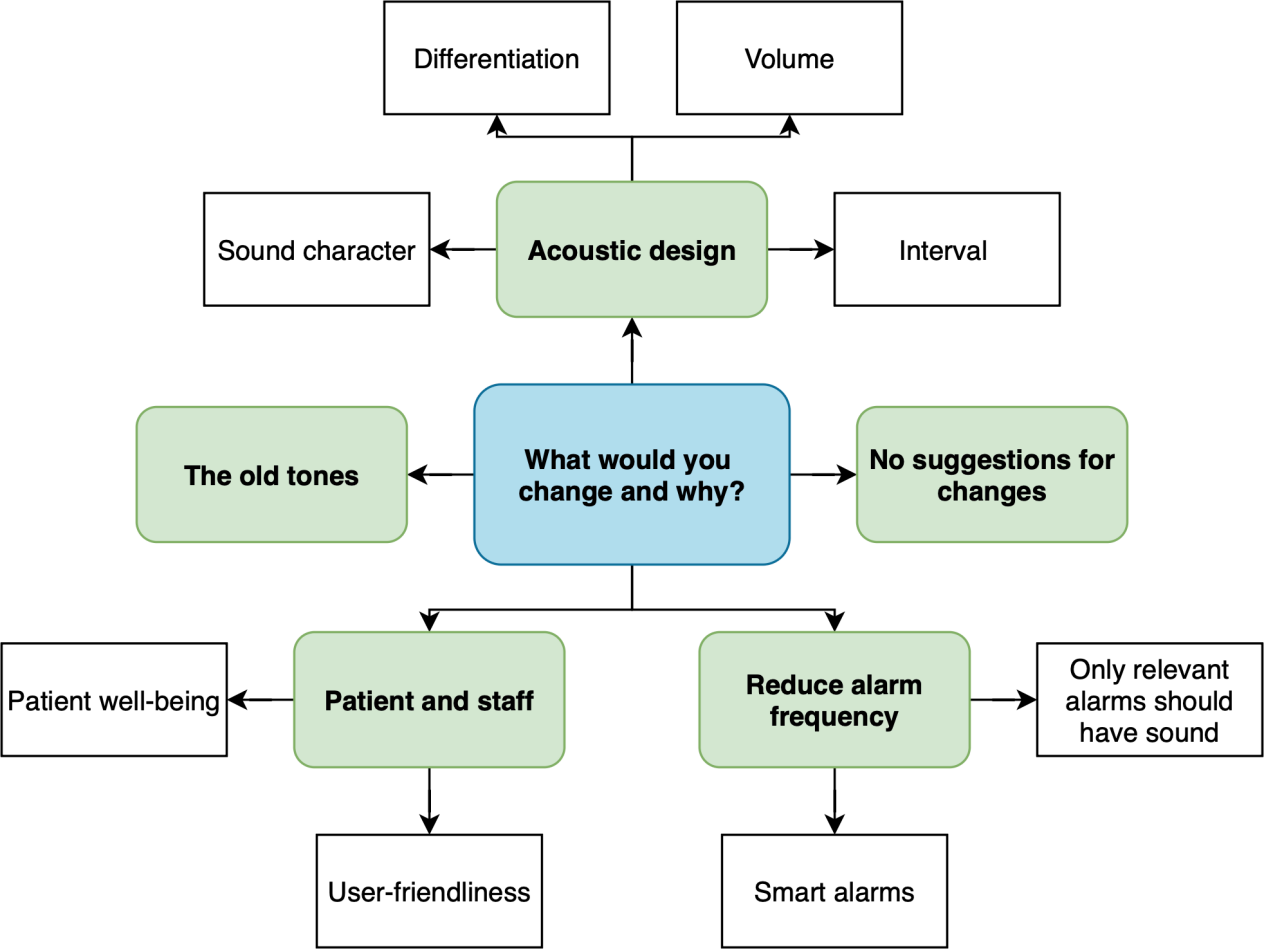
Coding tree illustrating the main themes and subthemes identified when participants were asked what they would change about the current alarm sounds and why.

**Figure 3.**
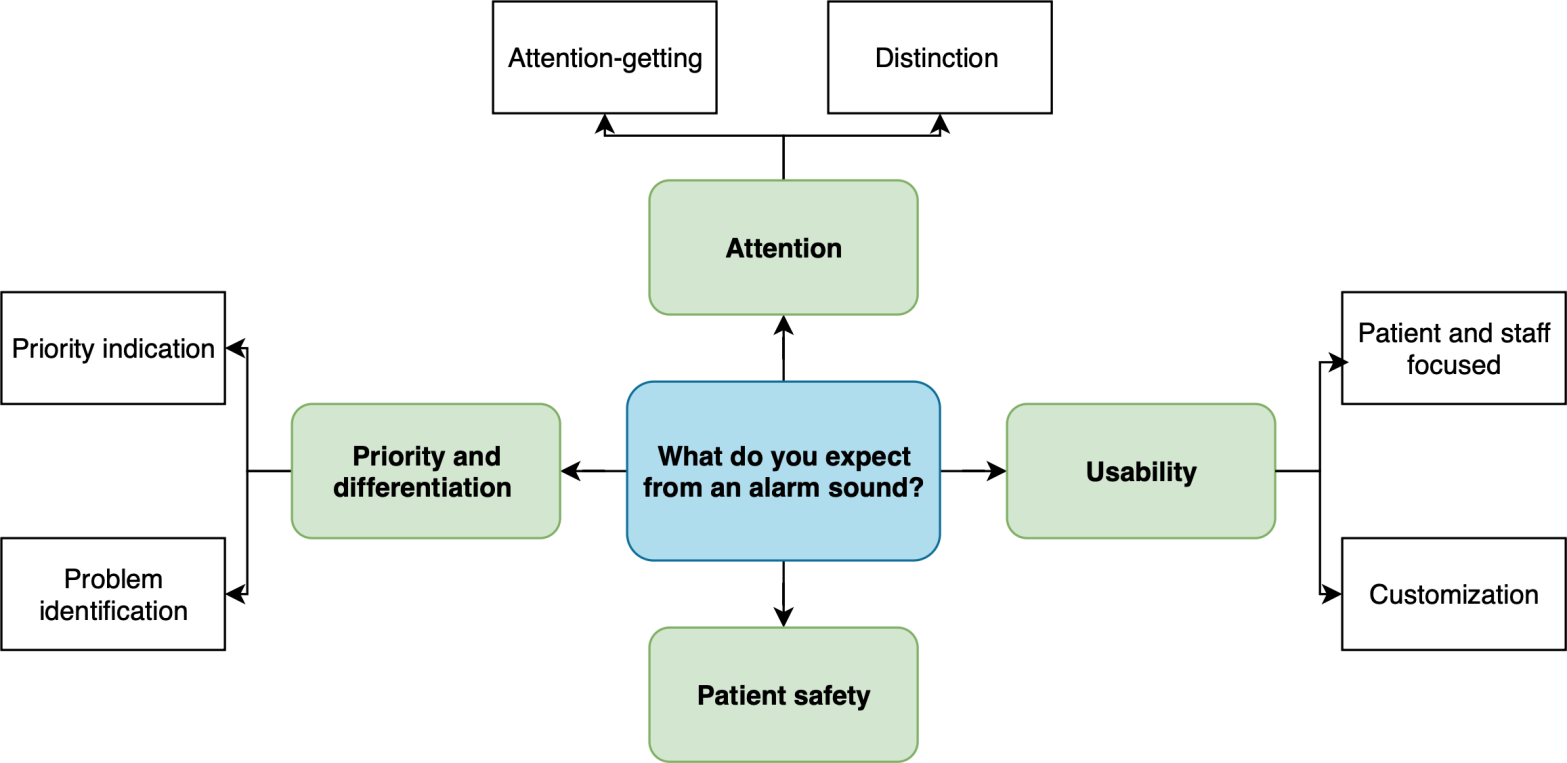
Coding tree illustrating the main themes and subthemes identified when participants were asked what they expected from an alarm sound.

**Figure 4.**
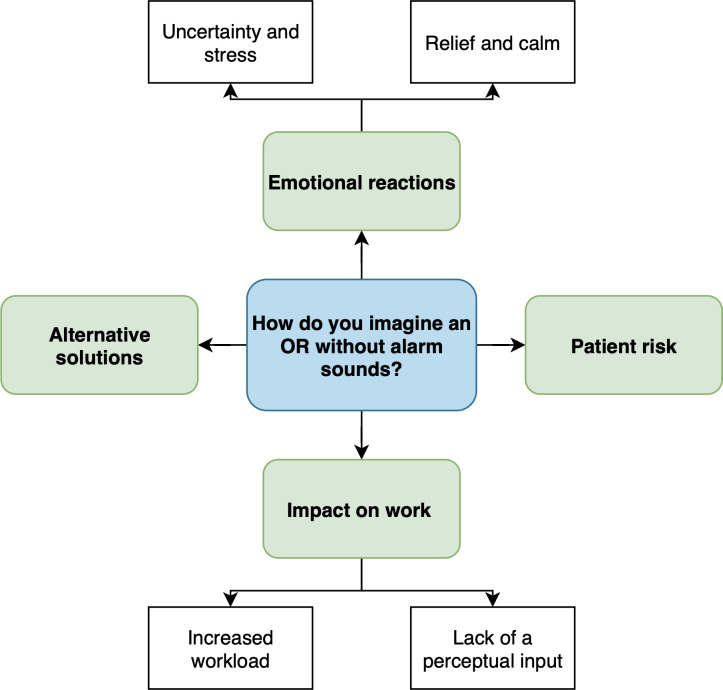
Coding tree illustrating the main themes and subthemes identified when participants were asked how they imagined an operating room (OR) without alarm sounds.

A 6-step thematic analysis method was used to examine the open-ended responses and identify dominant themes [21]. After thoroughly reviewing the data, the research team convened to share impressions and explore potential thematic concepts. On the basis of this discussion, 4 coding templates were developed for each question to facilitate further analysis (Figures 1-4). Participants’ responses were independently coded by 3 team members (GG, SS, and TRR). Any discrepancies in coding were resolved by the third coder (GG).

Closed-ended responses were analyzed using Microsoft Excel and presented as numerical values with percentage distributions or as medians with IQRs and minimum and maximum values.

## Results

### Participant and Study Characteristics

Of the 90 anesthesia providers initially contacted, all (n=90, 100%) completed the questionnaire during the prephase. In the postphase, 85.6% (77/90) of the potential participants submitted the questionnaire, as 14.4% (13/90) were unavailable due to a change in employment. A total of 888 comments were collected: 509 (57.3%) during the prephase and 379 (42.7%) during the postphase. A detailed study and participant characteristics are provided in Table 1.

**Table 1.** Participant and study characteristics.

	Values	
	Prephase (n=90)^a^	Postphase (n=77)
Participants who completed the questionnaire, n (%)	90 (100)	77 (100)
Collected comments, n	509	379
Age (y), median (IQR; range)	34 (30-41; 23-61)	34 (30-42; 24-62)
Sex, n (%)		
Female	45 (50)	34 (44.2)
Male	45 (50)	43 (55.8)
Experience (y), median (IQR; range)	5 (2.3-11; 0-40)	6 (2.8-12.5; 0-35)
Role, n (%)		
Nurse anesthetist in training	5 (5.6)	2 (2.6)
Certified nurse anesthetist	23 (25.6)	22 (28.6)
Resident—1-2 y of training	16 (17.8)	14 (18.2)
Resident—3-5 y of training	17 (18.9)	12 (15.6)
Resident—>5 y of experience	8 (8.9)	6 (7.8)
Staff anesthesiologist	21 (23.3)	21 (27.3)
“I play a musical instrument regularly (once or more per week),” n (%)	13 (14.4)	—^b^
“I attended the introductory lecture,” n (%)	39 (43.3)	—

a A total of 90 participants were contacted in both phases.

b The question was not repeated in the postphase.

### Main Themes From Responses to Open-Ended Questions: Impressions Before and After Implementing the Updated Philips Patient Monitor Alarm Sounds

#### Overview

Table 2 presents the themes and subthemes corresponding to the coding trees (Figures 1-4), as identified in participants’ responses, along with illustrative examples and their numerical and percentage distributions.

**Table 2.** Identified themes and subthemes in participants’ responses with examples, numerical values, and percentage distributions.

Theme and subthemes	Prephase comments, n/N (%)	Postphase comments, n/N (%)	Examples	
			Prephase (original alarms)	Postphase (updated alarms)
Question: “How do you feel about the current alarm sounds?”				
Emotional reactions				
Positive	37/139 (26.6)	46/89 (51.7)	“I am satisfied with the current alarm sounds as they help me alert to dangerous situations.” [Participant 72]	“I feel less stressed with the new alarm sounds.” [Participant 85]
Neutral	21/139 (15.1)	3/89 (3.4)	“Familiar alarm sounds that are well integrated into my daily workflow.” [Participant 46]	“I have gotten used to it.” [Participant 55]
Negative	30/139 (21.6)	7/89 (7.9)	“The more frequently and longer they sound, the more it increases my stress level while working.” [Participant 22]	“I am familiar with the old sounds and prefer them.” [Participant 73]
Perception of alarm sounds				
Acoustics	9/139 (6.5)	2/89 (2.2)	“The sounds are very high-pitched.” [Participant 27]	“The new sounds are more high-pitched, otherwise similar to the previous ones.” [Participant 22]
Frequency	10/139 (7.2)	0/89 (0)	“High-priority alarms sound too often.” [Participant 85]	—^a^
Functionality of alarm sounds				
Good functionality	9/139 (6.5)	9/89 (10.1)	“I perceive them as a high level of security, and they allow me to focus on the surgical tasks as well.” [Participant 38]	“The new alarm sounds capture appropriate attention.” [Participant 75]
Inadequate functionality	16/139 (11.5)	20/89 (22.5)	“The alarm sounds with ’low priority’ are often not noticed.” [Participant 87]	“I find that the new alarm sounds often go unnoticed in hectic situations.” [Participant 84]
Patients				
Patient dynamics	5/139 (3.6)	2/89 (2.2)	“I think that the alarm sounds are very unsettling for the patients.” [Participant 66]	“For the patients, the new alarm sounds are certainly much more pleasant and less startling.” [Participant 3]
Suggestions for improvement				
Acoustics	1/139 (0.7)	0/89 (0)	“The alarm sounds could be slightly lower in pitch.” [Participant 24]	—
Question: “What would you change and why?”				
No suggestions for changes^b^	26/112 (23.2)	22/84 (26.2)	“I would not change anything. The alarms fit into my workflows.” [Participant 75]	“I do not want to change anything.” [Participant 90]
Acoustic design				
Sound character	26/112 (23.2)	7/84 (8.3)	“I would wish for softer tones. The saturation tone drops too quickly to a lower pitch. From 100% to 98%, it startles me.” [Participant 33]	“The new alarm sounds are more pleasant.” [Participant 82]
Differentiation	11/112 (9.8)	27/84 (32.1)	“Alarms that indicate high priority should stand out more from the ’standard tones.' A better differentiation for emergencies to quickly grab the attention of the team.” [Participant 46]	“Low- and medium-priority alarm sounds are difficult to distinguish.” [Participant 70]
Volume	5/112 (4.5)	4/84 (4.8)	“The volume should gradually increase.” [Participant 29]	“Low-level alarms could be louder and more attention-grabbing.” [Participant 65]
Interval	10/112 (8.9)	6/84 (7.1)	“For lower alarm priorities, the frequency should be lower.” [Participant 6]	“I would like to increase the frequency of the middle tone a little.” [Participant 89]
Reduction of alarm frequency				
Smart alarms	7/112 (6.3)	2/84 (2.4)	“Filter settings and auto-limits; more ’intelligence’ in the alarm sounds.” [Participant 20]	“Reduce the Apnoea alarm during anaesthesia induction from high alarm priority to medium priority.” [Participant 29]
Only relevant alarms should have sound	17/112 (15.2)	8/84 (9.5)	“Most alarms are artifacts or clinically irrelevant.” [Participant 71]	“I only need two alarm priorities; technical alarms (low priority) are unnecessary.” [Participant 52]
Patient and staff				
Patient well-being	6/112 (5.4)	2/84 (2.4)	“For patients in the induction phase, the alarm sounds are very startling and sometimes frightening.” [Participant 51]	“Some patients found the sound unpleasant and asked if it would be heard the entire time.” [Participant 53]
User-friendliness	4/112 (3.6)	3/84 (3.6)	“Easily acknowledgeable alarms.” [Participant 22]	“I would like a simpler way to silence the alarm tone.” [Participant 46]
The old tones^b^	—	3/84 (3.6)	—	“The old alarm tones.” [Participant 53]
Question: “What do you expect from an alarm sound?”				
Attention				
Attention grabbing	49/134 (36.6)	46/104 (44.2)	“A good alarm tone grabs attention and does not allow for ignoring.” [Participant 5]	“Direct attention to the monitor and to the patient.” [Participant 22]
Distinction	22/134 (16.4)	11/104 (10.6)	“A good alarm tone must be easily distinguishable from other sounds.” [Participant 14]	“Important alarms should stand out. A low tone is distracting.” [Participant 2]
Priority and differentiation				
Priority indication	18/134 (13.4)	15/104 (14.4)	“I expect a simple classification of the alarm’s importance (i.e., distinction of priority).” [Participant 66]	“The ability to assess the alarm priority without directly looking at the monitor.” [Participant 4]
Problem identification	14/134 (10.4)	12/104 (11.5)	“I expect a precise differentiation of the problem.” [Participant 81]	“The corresponding meaning of the tone and its consequences are important.” [Participant 5]
Usability				
Patient and staff focused	24/134 (17.9)	17/104 (16.3)	“It should convey the information but be pleasant.” [Participant 31]	“The alarm tones should fulfil their purpose and not create additional stress.” [Participant 87]
Customization	5/134 (3.7)	2/104 (1.9)	“Alarm only when there is a real clinical emergency. Do not alarm for any technical issues.” [Participant 40]	“Alarm tones should be adjustable to the individual patient.” [Participant 3]
Patient safety^b^	2/134 (1.5)	1/104 (1.0)	“Safety for the patient.” [Participant 33]	“Patient safety.” [Participant 3]
Question: “How do you imagine an operating room without alarm sounds?”				
Emotional reactions				
Uncertainty and stress	49/124 (39.5)	37/102 (36.3)	“It would be a very large burden for me.” [Participant 38]	“It is not possible; it cannot be done.” [Participant 26]
Relief and calm	29/124 (23.4)	19/102 (18.6)	“A more pleasant work atmosphere where one can stay focused for longer periods.” [Participant 46]	“A calm, pleasant working atmosphere.” [Participant 3]
Impact on work				
Increased workload	8/124 (6.5)	10/102 (9.8)	“It is exhausting for anaesthesiologists, as the monitor must be constantly checked.” [Participant 66]	“It would make everyday tasks significantly more difficult, as it would require more frequent visual checks of the vital parameters, potentially preventing full concentration on other tasks.” [Participant 84]
Lack of a perceptual input	16/124 (12.9)	6/102 (5.9)	“I am used to working a lot with my hearing, and it helps in an emergency to quickly assess and focus on the patient.” [Participant 43]	“No focus because you would have to constantly look at the monitor.” [Participant 62]
Alternative solutions^b^	4/124 (3.2)	6/102 (5.9)	“Visual alarms, colours on the monitor.” [Participant 42]	“Further exploration/research into new ways of presenting alarms (e.g., tactile/visual) would definitely be advisable, to possibly achieve this in the near or somewhat distant future. It would certainly be desired.” [Participant 68]
Patient risk^b^	18/124 (14.5)	24/102 (23.5)	“However, patients would likely be at risk.” [Participant 11]	“I believe that would pose a risk to patient safety; I consider the alarm tones essential for staying focused, especially during long surgeries.” [Participant 89]

a Not applicable.

b Distinct themes without subthemes.

#### How Do You Feel About the Current Alarm Sounds?

We identified 2 main themes in participants’ responses: emotional reactions and alarm sounds’ functionality. Before the implementation of the updated Philips patient monitoring alarm sounds, 26.6% (37/139) of the collected statements reported a positive emotional response. After the update, this proportion increased to 51.7% (46/89), indicating a notable improvement in the perceived emotional acceptance of alarm sounds. However, concerns about the functionality of alarm sounds remained prevalent across both phases, with related issues reported in 11.5% (16/139) of preimplementation responses and 22.5% (20/89) of postimplementation responses, making it the second most frequently mentioned theme.

#### What Would You Change and Why?

The main themes identified in participants’ responses differed slightly between the pre- and postimplementation phases. In the prephase, the most frequently mentioned themes were no suggestions for changes (26/112, 23.2%), sound character (specifically the need to reduce high-pitched tones and make sounds softer and more harmonious; 26/112, 23.2%), and a suggestion to present only high-priority alarms with sound while representing low-priority alarms visually (17/112, 15.2%).

In the postphase, the dominant theme was the difficulty in distinguishing alarm sounds based on priority (27/84, 32.1%), highlighting the need to make tones for different priority levels (low, medium, and high) more distinct. The second most frequently mentioned theme, as in the prephase, was that no changes were required (22/84, 26.2%).

#### What Do You Expect From an Alarm Sound?

The responses in the pre- and postimplementation phases differed slightly. In both phases, expectations focused primarily on an alarm sound’s ability to capture attention (49/134, 36.6% in the prephase vs 46/104, 44.2% in the postphase), emphasizing the need for effectiveness even in noisy environments. The second most frequently mentioned theme in both phases was priority indication (18/134, 13.4% in the prephase vs 15/104, 14.4% in the postphase), meaning that an alarm sound should clearly convey the urgency of a situation.

In the prephase, another dominant theme was the distinction of alarm sounds from other sounds (22/134, 16.4%). In contrast, in the postphase, a new theme emerged: usability in terms of alarms addressing both staff and patient needs (17/104, 16.3%), meaning that they should be easily perceptible for staff without causing stress for patients.

#### How Do You Imagine an Operating Room Without Alarm Sounds?

The same themes dominated participants’ responses to this question: uncertainty and stress due to increased effort and strain from the lack of auditory cues (49/124, 39.5% in the prephase vs 37/102, 36.3% in the postphase), relief and calm associated with reduced noise and a more pleasant work environment (29/124, 23.4% in the prephase vs 19/102, 18.6% in the postphase), and patient risk as the absence of alarm sounds may lead to overlooked critical situations or delayed reactions to changes (18/124, 14.5% in the prephase vs 24/102, 23.5% in the postphase).

## Discussion

In this investigator-initiated, single-center qualitative descriptive survey study, we explored anesthesia providers’ perceptions of patient monitoring alarm sounds before and after the clinical introduction of the redesigned Philips alarms.

While the updated alarm sounds were associated with a greater proportion of positive emotional responses among the study participants, the perceived issue of clearly distinguishing between alarm priorities persisted. Before the update, participants predominantly reported difficulties in differentiating between medium- and high-priority alarms. However, after the update, they indicated increased difficulty distinguishing between low- and medium-priority alarms.

Alternative strategies, such as user training for alarm threshold adjustment; reduction of overmonitoring [2,22]; and advanced technical solutions, such as smart alarms, multiparameter integration, and short-delay algorithms, have shown greater promise in reducing alarm fatigue than alarm design alone [23]. In addition, innovative acoustic approaches, including auditory icons, spearcons, and voice alerts, may further enhance alarm systems. Studies suggest that these formats improve speed and accuracy of interpretation while lowering cognitive load [9,24-26]. Importantly, the International Electrotechnical Commission (IEC) explicitly recommended the use of auditory icons in its revised global standard for medical equipment alarms (IEC 60601-1-8) published in 2020 [27].

Beyond acoustic design, the ergonomics of the patient bedside are also important. Limited visibility of alarm triggers may lead clinicians to rely on multiple auditory cues from multiple devices. Optimizing screen layout, integrating networked devices with hierarchical alarms, and using smart display strategies such as a patient avatar could reduce cognitive load and improve timely responses [28]. These ergonomic and acoustic considerations provide a useful framework for understanding how clinical staff actually perceive and interact with alarm sounds in practice.

In our study, participants primarily perceived the role of acoustic alarms as drawing attention to the patient monitor rather than conveying specific clinical information. It is noteworthy that participants did not suggest innovative ways in which acoustic alarms could communicate specific deviations in vital signs. Similarly, established alternative sonification approaches, as discussed in the literature [29] and reflected in the IEC 60601-1-8 international standard, were not referenced in the responses. This may suggest limited awareness or perceived relevance in the clinical context [27]. This observation may be explained by the innovation paradox inherent to user-centered design: users tend to articulate needs and preferences based on familiar concepts and experiences [17]. Thus, if design processes rely exclusively on user feedback, opportunities for more radical or transformative innovations may be overlooked. Therefore, to foster meaningful advancements in alarm systems, user-centered design should be complemented with expert-driven innovation strategies and evidence-based frameworks.

This study also explored the radical notion of eliminating acoustic alarms altogether. Surprisingly, only a small proportion of participants considered this to be a significant risk to patient safety, despite the fact that acoustic alarms are mandated as essential patient safety tools in various guidelines and industry standards [1,4,7]. This finding underscores the need for ongoing education about the safety functions of alarms and the complex trade-offs between alarm fatigue and patient risk.

One particularly interesting and easily implementable idea was repeatedly mentioned by participants: anesthesia providers suggested eliminating the auditory presentation of low-priority alarms, opting instead for visual-only indications. A closer examination reveals that low-priority alarms in Philips patient monitors predominantly signal technical issues unrelated to the patient’s clinical status and typically do not require intervention of the caregiver. We believe that, depending on the clinical context—whether intensive care units, operating rooms, recovery rooms, or telemetry wards—the function and role of alarms vary significantly. Different rooms, different rules: what works in the operating room may not suit intensive care. User feedback is invaluable for evaluating these different use profiles. However, any changes to alarm management strategies, particularly the reduction of acoustic cues, must be carefully evaluated against existing safety guidelines and subjected to thorough risk assessments to ensure that patient safety is not compromised.

This study has several notable strengths. It was conducted in a real-world clinical environment at a large tertiary university hospital, enhancing the external validity and practical relevance of the findings [30]. The pretest-posttest design allowed for a direct comparison of perceptions before and after the implementation of the updated Philips patient monitoring alarms [31]. Furthermore, open-ended responses were systematically analyzed using a well-established 6-step thematic analysis method [21], with independent coding by multiple researchers to ensure analytical rigor and minimize bias. The provision of original study materials, including the questionnaire and multimedia supplements, further strengthens the transparency and reproducibility of the research.

Nonetheless, several limitations must be acknowledged. The single-center pretest-posttest design without a control group precludes causal inference, does not account for time-dependent effects, and limits generalizability [30]. However, the within-subject design, standardized training, and extended adaptation period were chosen to enhance internal and ecological validity for the evaluation of alarm sound perception in a real-world clinical setting. Participation was voluntary, which may have introduced selection bias, as individuals with greater interest in alarm systems or new technologies might have been more likely to participate. The questionnaire, although carefully designed, was not externally validated, potentially affecting the reliability of the findings. It should also be noted that while the patient monitor was evaluated in a clinical setting in the operating room, the study did not assess the monitor within the full operating room soundscape, which may influence real-world perception. Finally, perceptions were self-reported and, therefore, subject to biases such as social desirability or recall bias. Moreover, while improvements in emotional acceptance of alarm sounds were achieved, it is critical to emphasize that positive emotional responses alone do not guarantee enhanced patient safety. Functional effectiveness, particularly the rapid and accurate identification of alarm priorities, remains paramount.

Despite these limitations, this study provides valuable insights into the user experience with updated patient monitoring alarms and highlights important considerations for future alarm design, development, and evaluation efforts. When developing new technological solutions to reduce alarm frequency and false positives and improve the distinguishability and intuitive meaning of alarm signals, it is crucial not to rely solely on user-centered design [17]. Current evidence [9,24,25], international standards [27], and expert input must also be incorporated to achieve an optimal balance among usability, safety, and innovation. Future patient monitoring alarm systems could benefit from a multimodal approach that integrates conventional alarm sounds with auditory icons, voice alerts, and advanced visual displays to better support clinical decision-making and effectively mitigate alarm fatigue for both caregivers and patients [29].

## Supplementary material

10.2196/82703 Multimedia Appendix 1 Original and updated Philips patient monitoring alarm sounds.

10.2196/82703 Multimedia Appendix 2 Data generated and analyzed during this study.

## Abbreviations

IEC: International Electrotechnical Commission
