# Supplementary material for: Anesthesia Providers’ Perspectives on the Redesigned Philips Acoustic Alarm System: Qualitative Pre- and Postimplementation Study
Source: JMIR Hum Factors. 2026 Apr 1;13:e82703. doi: 10.2196/82703 (PMC13041626; doi:10.2196/82703)
Supplement: Multimedia Appendix 1 [file humanfactors-v13-e82703-s001.pptx]

## Slide 1
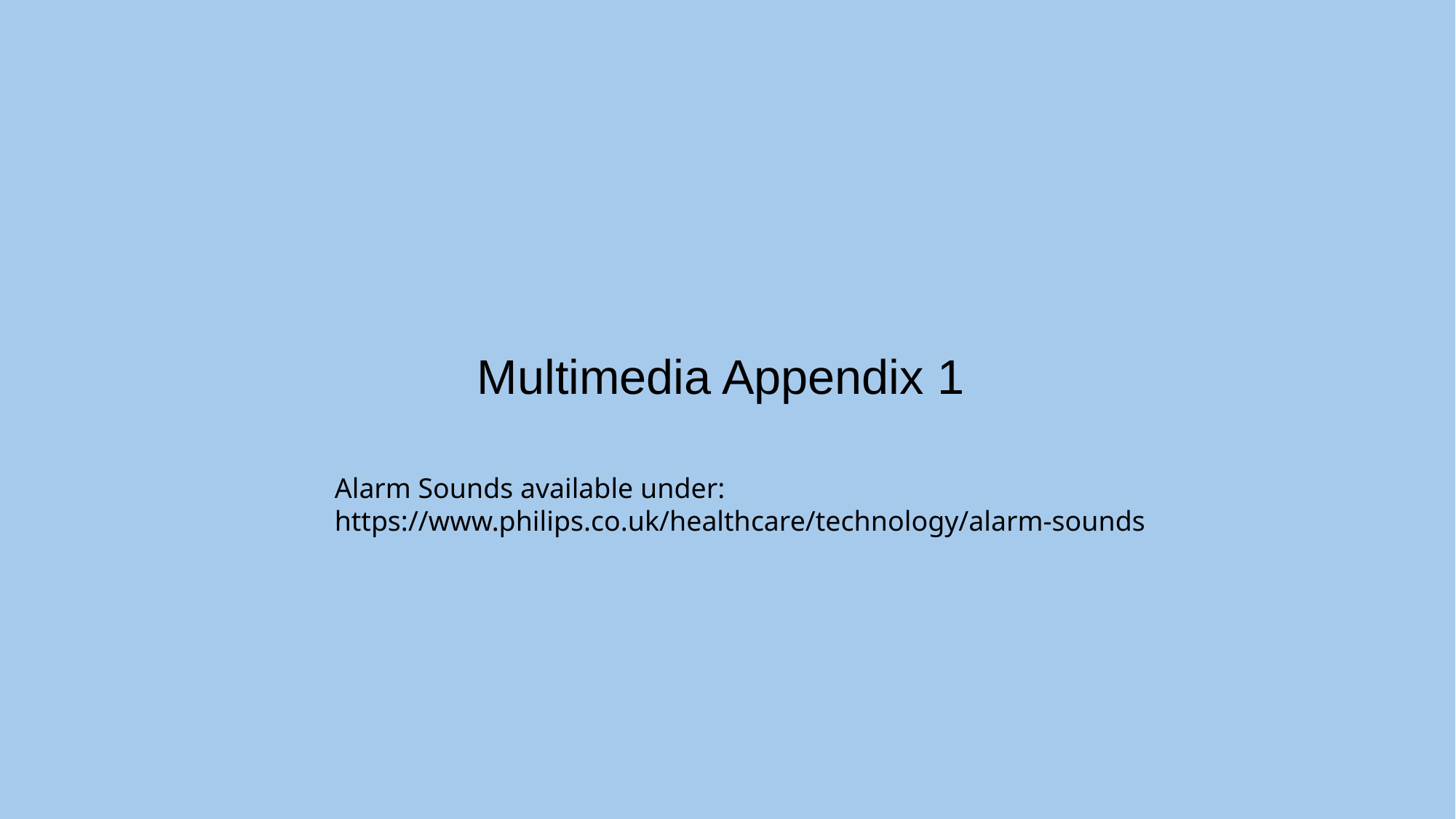

# Multimedia Appendix 1
Alarm Sounds available under:
https://www.philips.co.uk/healthcare/technology/alarm-sounds

## Slide 2
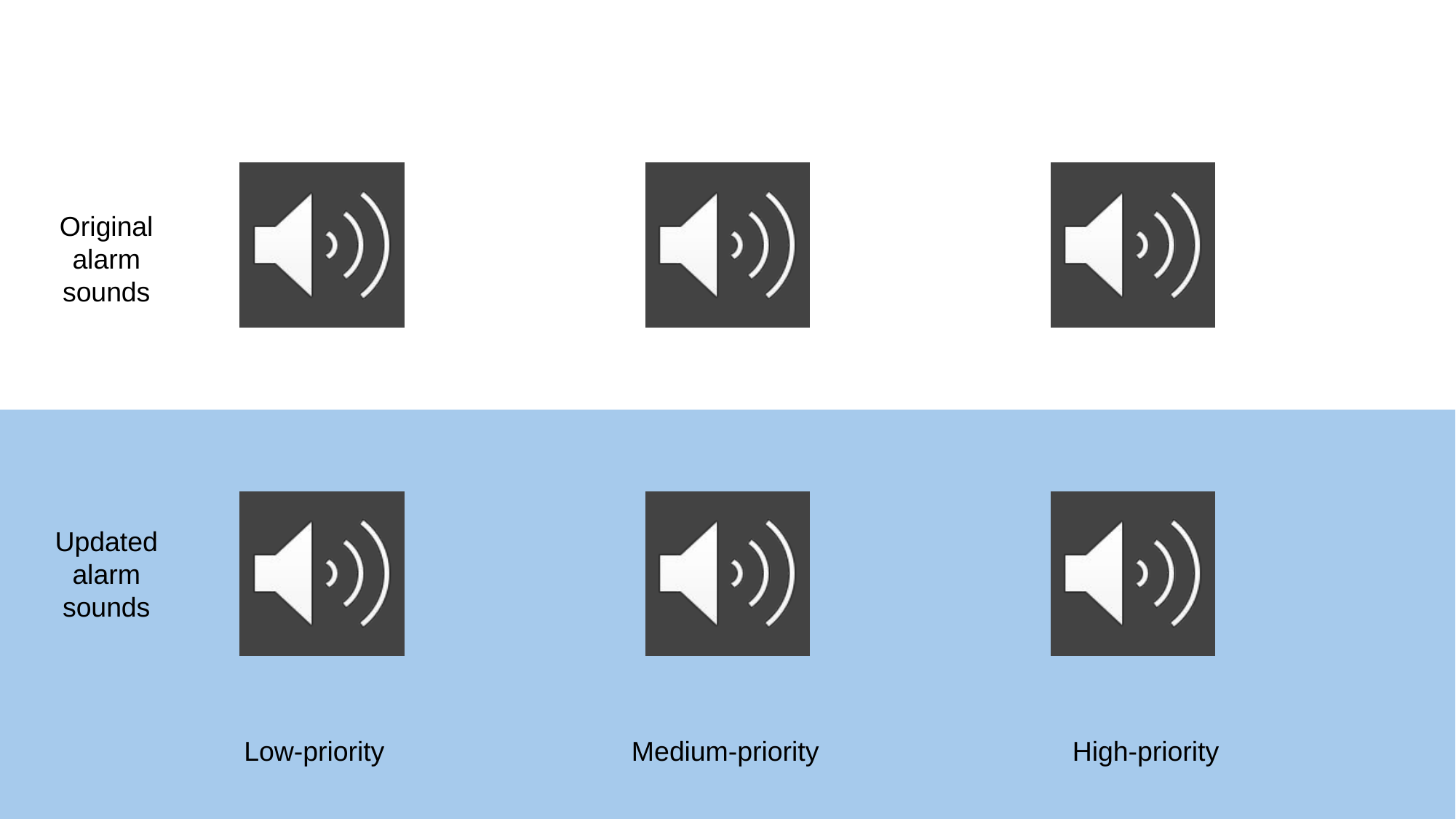

Original alarm sounds
Updated alarm sounds
Low-priority
Medium-priority
High-priority
